# Surfactants, Nanomedicines and Nanocarriers: A Critical Evaluation on Clinical Trials

**DOI:** 10.3390/pharmaceutics13030381

**Published:** 2021-03-13

**Authors:** Diego Alejandro Dri, Carlotta Marianecci, Maria Carafa, Elisa Gaucci, Donatella Gramaglia

**Affiliations:** 1 Clinical Trials Office, Italian Medicines Agency (AIFA), Via del Tritone 181, 00187 Rome, Italy; e.gaucci@aifa.gov.it (E.G.); d.gramaglia@aifa.gov.it (D.G.); 2 Department of Chemistry and Technology of Drugs (DCTF), University of Rome “La Sapienza”, Piazzale Aldo Moro 5, 00185 Rome, Italy; maria.carafa@uniroma1.it

**Keywords:** clinical trials, nanomedicines, nanocarriers, regulatory, surfactants

## Abstract

Advances, perspectives and innovation in drug delivery have increased in recent years; however, there is limited information available regarding the actual presence of surfactants, nanomedicines and nanocarriers in investigational medicinal products submitted as part of a request for authorization of clinical trials, particularly for those authorized in the European Economic Area. We retrieve, analyze and report data available at the Clinical Trial Office of the Italian Medicines Agency (AIFA), increasing the transparency and availability of relevant information. An analysis of quality documentation submitted along with clinical trials authorized by the AIFA in 2018 was carried out, focusing on the key terms “surfactant”, “nanomedicine” and “nanocarrier”. Results suggest potential indications and inputs for further reflection and actions for regulators to actively and safely drive innovation from a regulatory perspective and to transpose upcoming evolution of clinical trials within a strong regulatory framework.

## 1. Introduction

Recent publications and reviews have illustrated the future of nanomedicines and nano-based drug delivery systems [1]. Therapeutic and diagnostic nanocarriers typically fall into two categories: (a) inorganic nanoparticles (e.g., gold, silica, iron oxide) and (b) organic nanoparticles (e.g., polymeric, liposomes, micelles). Inorganic nanoparticles have been successfully applied in clinics for diagnostic purposes, while organic nanoparticles show a broad application in therapy, ranging from vaccination to tumors. The selection of the material of nanocarriers is a function of the desired therapeutic or diagnostic goals, of the route of administration, of the safety profile of the material and of the nature of the active molecule they have to entrap [2,3].

**Figure 1:**
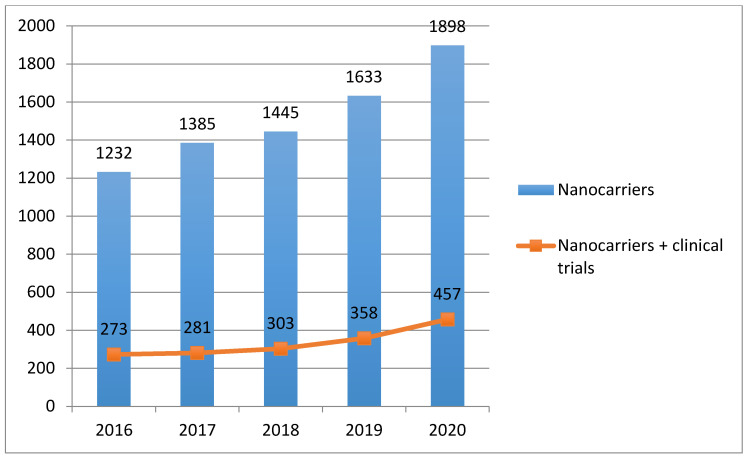
Trend in number of publications containing the term “nanocarriers” (histogram), and in number of publications containing the term “nanocarriers + clinical trials”, in the last 5 years (searching criteria: article title, abstract, keywords). Source: www.scopus.com (accessed on 6 February 2021).

The advances in formulation development, characterization, testing and non-clinical safety evaluation of delivery systems of chemical entities such as non-ionic surfactant-based nanocarriers (niosomes, nanoemulsions, micelles, etc.) have received ever-increasing attention [4]. Potential innovation of nanotherapeutics in drug delivery and application of nanotechnology including perspectives in the fields of nanorobots, nano-diagnostics and personalized medicine [5] are highlighted. The interest of researchers in nanocarriers has been constantly growing in recent years and this trend, resulting in more than 7500 publications in the last 5 years, can be easily verified with the Scopus search engine, and interesting, indeed, is also the trend in publications considering the documents by year retrieved searching for the combination of terms “nanocarriers” and “clinical trials” (Figure 1).

However, how many of these innovations actually find a real application in the clinical development of a medicinal product or device and are granted a marketing authorization? The general conclusion is that translation of scientific research into a clinical application represents a major challenge, where the main and critical issue is the toxicity and safety evaluation. Other manuscripts highlight, in fact, how the quality by design approach would be envisaged for the development of nanopharmaceutical products in order to limit risks due to high variability in quality [6,7]. A modern formulation approach to the design of experiments has also been reported, but again, in this case, it is, at the same time, highlighted that translation of certain nanomedicines from the laboratory to market has been very limited [8]. The clinical translation of nanomedicines is an expensive and time-consuming process, more complex in comparison to conventional formulation technology. The time needed to reach a clinical translation is relatively long and the path is very costly and complex. These facts strongly affect the attitudes of the pharmaceutical industry and capital investors. In particular, the cost–benefit analysis may be a limitation to the clinical translation of some nanomedicines when compared to an approved counterpart or existing therapies; nevertheless, nanopharmaceuticals can offer the possibility to prolong the economic life of proprietary drugs [9].

In order for a medicinal product to be granted a marketing authorization according to the governing rules in the European Union (EU), the application dossiers submitted by pharmaceutical companies applying for a marketing authorization must include and rely on the results of the clinical trials (CTs) on medicinal products for human use that are submitted in the clinical study reports section, which is a large part of the application. CTs are studies that are intended to discover or verify the effects of one or more investigational medicines and need to follow specific rules and guidelines in the EU [10].

Conducting CTs is the crucial step to determine the safety and efficacy of investigational medicinal products (IMPs), and the key driver of medical innovation and progress in patient care and disease prevention. The quality assessors at the national competent authorities (NCAs) would be the first ones in assessing innovative ways to transport the drug into the target and would be facing and dealing with the assessment of the most recent and ultimate scientific innovations regarding IMPs. The evaluation of data submitted by the sponsors within an application requesting authorization of a CT is expected to provide, at a glimpse, a snapshot showing to what extent the latest and innovative advances in pharmaceutical nanotechnology, including surfactants, nanomedicines and nanocarriers, are actually currently used in IMPs and are therefore candidates to be, in the near future, part of a marketing authorization application.

It is, on the other hand, acknowledged that there are limited guidelines available, particularly in the context of a CT evaluation, to support quality, safety and efficacy assessments in the field of nanotechnology-enabled health products, encompassing within this term nanomedicines and nanomedical devices. On this topic, a white paper discussing how regulatory science can react and advance was recently published by the Joint Research Centre (JRC), the European Commission’s (EC) science and knowledge service [11]. However, even if a definition of nanomedicine is not currently standardized and acknowledged worldwide, in the EU, it is defined as the application of nanotechnology in view of making a medical diagnosis or treating or preventing diseases, and it exploits the improved and often novel physical, chemical and biological properties of materials at nanometric scale [12]. A working definition for the plural term nanomedicines, as purposely designed systems for clinical applications with at least one component at nanoscale size and resulting in definable specific properties and characteristics, is also available [13]. Other terms are currently arbitrarily used, such as nanostructure, nanoparticle or nanomaterial; we therefore make reference to nano-related terms according to the mapping of nanomedicine terminology in the regulatory landscape of the JRC [14]. We also take into account the overview of concepts and terms used in the definition of nanomaterial [15] in a regulatory context, categorizing a nanomaterial if 50% or more of its constituent particles fall in the size range from 1 to 100 nm.

The European Medicines Agency’s (EMA) “Regulatory Science to 2025” strategic reflection publication [16] clearly identified in the EU as strategic regulatory science goals, among others, the following: develop understanding of, and regulatory response to, nanotechnology and new materials in pharmaceuticals; raise awareness of new nanomedicines and materials via the EU Innovation Network; generate guidance addressing PK/PD requirements and long-term efficacy and safety; enable and leverage research and innovation in regulatory science; foster innovation in CTs.

When performing a bibliographic research in order to identify publications on the use of surfactants, nanomedicines and nanocarriers in CTs, it was realized that there is actually very limited information and, when this is available, reference is made to CTs authorized by the FDA and that the information is mainly retrieved from ClinicalTrials.gov [17].

The CT application form (CTA) in the Annex 1 [18] of the detailed guidance [19] of the EC on the request to the NCAs for authorization of a CT does not envisage as structured data in section “D Information on each IMP” the identification of a nanomedicine or a nanodevice and its characteristics; if the composition of the IMP includes nanocarriers; or if, as a consequence of the specific formulation of the IMP, the potential use of surfactants, any nanostructure or nanomaterial is involved. However, this information would be relevant for the purpose of identifying pharmacokinetic, efficacy, safety and targeting benefits [20] derived from peculiar physicochemical properties, and the availability of quality information could provide alerts and insights on potential toxicity issues. The lack of dedicated structured data prevents retrieving straightforward information through a query in the databases that contain information on CTs taking place in the European Economic Area (EEA) and clinical studies conducted worldwide in accordance with a pediatric investigation plan (EudraCT) [21], a subset of whose data is publicly accessible via the European Clinical Trials Register [22]. A query in NCA databases, such as the Osservatorio Nazionale delle Sperimentazioni Cliniche (OsSC) [23] in Italy, does not even support collecting this information, even though the OsSC represents, in the EU, a platform model for e-submission, workflow and databases on CTs and allows providing information to stakeholders that is published on a yearly basis.

Due to the lack of relevant structured data, an attempt to at least nearly identify the CTs where a surfactant, nanomedicine or nanocarrier is concerned may only be made by performing a specific query on free text search fields of the CT databases and registers publicly available. When searching for the specific terms such as “nano”, “surfactant” and “carrier”, we get a very limited, not exhaustive picture of their potential use in CTs, depending only and relying on the descriptive information compiled into the systems by sponsors during the submission of an application for a request of authorization of a CT. In addition, the majority of reviews and papers refer to information extracted from ClinicalTrials.gov [24,25,26]; meanwhile, limited publications are instead based on clinicaltrialsregister.eu [27], and, moreover, recent reviews of nanomedicines already on the market usually only refer to their FDA approval date [28].

To our knowledge, unfortunately, in none of the public registries of CTs currently available is it possible to clearly distinguish among CTs involving nano-related medicines or devices, surfactants or carriers. As a matter of fact, in order to extract more detailed and accurate information to identify CTs involving nanodrugs and nanodevices, another strategy was attempted, using an innovative machine learning (ML) approach [29] to automatically detect the presence of nanotechnology-based products in CT summaries, but again only referring to those available on ClinicalTrials.gov and performing text search browsing studies by keyword. However, this strategy would not have been effective and results accurate if using EudraCT and the EU Clinical Trials Register. The submission of the results to EudraCT is the direct responsibility of the sponsors [30], but 31.8% of the CTs as of April 2019 were not in compliance with the publication rules. Therefore, a Joint Letter by the European Commission, EMA and Heads of Medicines Agencies had to be issued in order to improve compliance on the posting of results, reminding all sponsors about their obligation to report CT summaries in the EU CTs database [31]. National databases for the management and submission of CTs are currently implemented and managed by some member states (MS) in the EEA, even though there is no possibility to retrieve from these the required information. Tight collaboration between the EMA and every single NCA in the EU is always necessary to build and maintain consolidated information on current CTs in all the EEA MSs; however, issues with national CT systems or different processes implemented nationally may lead to misalignments. This scenario should hopefully be improved by increasing the alignment across the EU upon the entry into application of the Regulation (EU) No 536/2014, thanks to the huge efforts that MSs are currently putting in place to support the EMA in developing the Clinical Trial Information System, also known as the EU portal and database, a unique system for submission and evaluation of CTs in the EEA.

We hereby retrieved and analyzed data on CTs authorized in Italy and available at the Clinical Trials Office of one NCA in the EU, discussing data and providing information on the current actual use of surfactants, nanomedicines and nanocarriers in the context of CTs submitted and authorized by the AIFA in 2018, thus providing a potential indication of their use across CTs in the EEA.

Further, we provide identification of inputs for further regulatory reflections and actions to support an active and safe driving of the innovation and upcoming evolution of CTs within a challenging and strong regulatory framework.

## 2. Materials and Methods

The process applied to obtain consistent and reliable information consisted in a manual assessment of every CT application, and, in particular, the quality documentation of the Chemistry Manufacture and Control (CMC) included in the Investigational Medicinal Product Dossier (IMPD), received as part of a CT application, was analyzed by retrieving the dedicated and updated documentation, opening and searching within every and each of the documents the key terms “surfactant”, “nanomedicine” and “nanocarrier”, as reported and declared by the sponsors.

The management of such a kind of data and information is critical, and it is mandatory to guarantee the protection of commercially confidential information; therefore, due to this reason, the full database used for this research and comprehensive quality information cannot be disclosed. It is anyhow important to take into consideration that already back in 2012, it was acknowledged that in order to increase transparency in the area of CTs, data submitted in support of a CT application should be based only on CTs recorded in a publicly accessible database [32], and this was indeed one of the bases for setting in 2014 the new Regulation (EU) No 536/2014.

The number of CTs that were submitted to the AIFA in 2018 was 714, and it is estimated that these totaled around 22.0% in relation to all CTs registered in the EudraCT system and conducted in the European Union in that year; therefore, an analysis of this set of data can definitely give a real and reliable potential indication of what the impact is globally across the EU.

At the time this article was written, at the Italian Medicines Agency Clinical Trial Office, a consolidated list of CTs authorized in 2018 was available, and related information was already made available through the “Rapporto Nazionale sulla Sperimentazione Clinica” [33]. This list was taken as a core dataset, and some additional specifications were defined to narrow the framework of our research. The analysis was carried out on the 666 CTs authorized in the AIFA in 2018, 433 of which included at least one IMP in the scope of the research, because the IMP to be used in the trial was declared not to have a marketing authorization.

Medicinal products with a marketing authorization are also considered as IMPs when they are included as a test substance or reference substance in a CT, provided they are formulated or packaged in a way different from the authorized form, or if they are used for the purpose of testing a different indication from the authorized one, or to gain further information (e.g., safety) about the authorized form. Reference products used as comparators should also be considered as IMPs. However, for the purpose of this research, we are interested in obtaining an overview of the use of potential new nanomedicines, IMP nanostructures or nanomaterials, surfactants and nanocarriers, investigated in CTs, and we therefore excluded those medicinal products based on active substances that had already been approved and for whom information and reviews are already available [34,35]. Phase IV CTs were accordingly excluded. Those IMPs that, even if already marketed, are declared in the CTA as not having a marketing authorization because they are actually being investigated for a different indication or with a different formulation [36], and therefore experimental batches are used, were instead taken into consideration in terms of the description and composition of the drug product. IMPs declared to be used as comparators or placebos were also excluded from the scope.

## 3. Results

### 3.1. Surfactants

In 199 out of 433 CTs (45.96%), we found that for the tested IMPs, in the composition of the drug product reported in the CMC section of the IMPD, at least one surfactant was listed.

Even if in most of the cases the excipient was listed with the function of surfactant, there were many other functions reported and associated with the surfactant, such as stabilizer, solubilizing agent or even not specified at all.

No topical formulation was identified containing surfactant, nanomedicine or nanocarrier in the IMPs authorized in 2018.

A self-microemulsifying drug delivery system (SMEDDS) was reported by sponsors in two oral formulations, developed to enhance the diffusion rate and oral bioavailability of IMPs.

The mixture of a drug substance, hydrophilic component, lipophilic component and surfactant spontaneously forms a microemulsion upon dilution with water, preventing the precipitation of the active substance. Immediate-release, self-emulsifying, semi-solid lipid formulations are contained in capsules, and they constitute an effective delivery vehicle with the potential for a much longer shelf life. SMEDDS were mainly discussed in terms of formulation development, bioavailability, dissolution in water, stability and excipient compatibility, and, in one case, a particle size distribution evaluation (0.1 to 100 μm) was also performed.

Polysorbates are the most common non-ionic surfactants used as stabilizers in protein formulations, reducing the rate of aggregation and precipitation when the protein is handled and agitated as a liquid. Polysorbates are indeed used to protect from stresses that may occur during processing (e.g., freezing and thawing) or handling.

**Figure 2:**
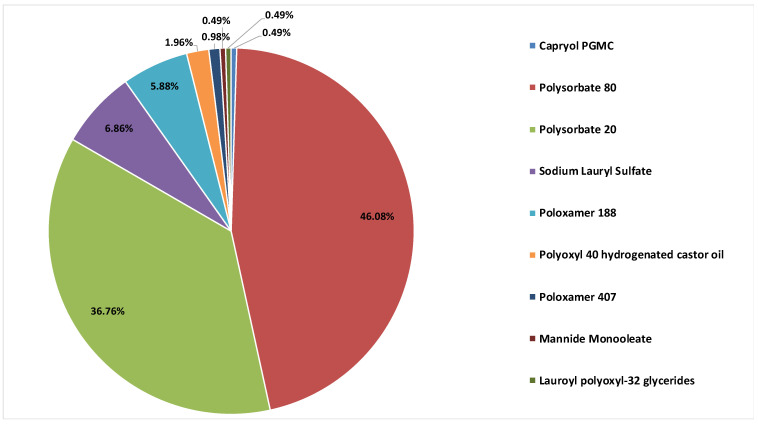
Percentage of instances for different surfactants found in the composition of the investigational medicinal products (IMPs).

Polysorbate 80 (46.08%) and polysorbate 20 (36.77%) were the standard surfactants used, together accounting for 82.85% of the instances, sodium lauryl sulfate (6.86%) and poloxamer 188 (5.88%) were only used to some extent, polyoxyl 40 hydrogenated castor oil was used in four instances, poloxamer 407 was used in two instances and, in only one instance each, lauroyl polyoxyl-32 glycerides, mannide monooleate and propylene glycol monocaprylate were also identified as surfactants used in the IMP formulation. Polysorbate is one of the most important non-ionic surfactants employed in surfactant-based nanocarrier formulations [4]; nevertheless, we were not able to retrieve any evidence or specific information potentially relating the use of a polysorbate as a component of a surfactant-based carrier in the formulation of IMPs, and consequently no dedicated characterization was available. We report in Figure 2 the percentage of instances for different surfactants found in the composition of the IMPs.

There is no evident correlation between the use of a specific surfactant and the phase of the study or the therapeutic area, this is compatible with a continuous development of the medicinal product and the attempts to define the proper final formulation that changes over time and across CT phases (Table 1).

Almost all CTs authorized at the Italian CA in 2018, accounting for 1/5 of all CTs conducted in the EU, are testing IMPs containing consolidated excipients utilized with a declared surfactant function, and the manufacture of the finished medicinal products practically does not include any novel excipient but rather well-known and accepted excipients in the manufacture of medicinal products for human use that comply with and are tested corresponding to the requirements of the respective pharmacopoeia monographs (surfactant chemical structure is reported in Table S1). This simply means that no pre-clinical evaluations and pharmacokinetics for a new excipient have to be completed to demonstrate its safety, no dedicated method has to be developed and validated and no information is needed to be provided on the manufacturing process, stability, characterization and control in relation to product safety. When consolidated surfactants are used, a potential micellar solution may not be fully described and the solubilizing capacity or properties of the medicinal product ingredients (e.g., function, relevant critical quality parameters affecting drug product performance and safety, physicochemical characterization, surfactant polydispersity and purity) may not actually be fully considered and justified in the IMPD. In case different kinds of surfactants (non-ionic, anionic and cationic surfactants) are combined, other properties such as the ionic strength or pH would also need to be discussed in the formulation justification. However, this is a cost-effective approach, facilitating and speeding up the clinical development of medicinal products but, at the same time, slowing down the application of new technologies and the testing of new potential molecules capable of being effective as drug delivery, increasing the opportunities to improve the treatment of diseases and increasing the chances of access to healthcare and innovative medicinal products. This may be indeed identified as a regulatory challenge [37], but the need for comprehensive safety tests analogous to the ones required for a new active substance should not be avoided and the solution should not be a shortcut in data availability supporting the safety profile.

### 3.2. Nanomedicines and Nanocarriers

Only 10 out of 433 CTs presented, in the CMC section of the IMPD, a specific reference to, or description of, a carrier.

Human serum albumin acting as a carrier protein, a synthetic peptide bound to a carrier protein, red blood cells, CRM197, mPEG succinimide and the tetanus toxoid protein were identified as carriers; in another case, the use of lactose α-monohydrate as a carrier to carry the active substance particles on its surface up to the moment of inhalation when the active substance de-aggregates and detaches from the surface carrier particles was identified, and the dosage form was an inhalation powder. Among solid oral dosage forms, instead, copovidone, sugar spheres and vitamin E polyethylene glycol succinate were also reported as carriers.

However, in none of these cases was the term “nanocarrier” explicitly used to describe the carrier, nor was it put in relation to the IMP as a nanomedicine, rather than making reference to a nanostructure or nanomaterial; however, for some of them, according to their characterization, results may be compatible with the nanometer scale.

It should be noted that these carriers were all employed in the manufacturing of IMPs for CTs at Phase II and Phase III, and it is not possible to identify any significative correlation for the use of carriers, as declared by sponsors, across therapeutic areas.

Even if no specific review was performed on the full list of excipients declared by sponsors for each and every IMP in the IMPDs submitted, as this was not in the scope of the research, it was, however, noted that some excipients were sometimes found to be listed but not employed as drug carriers, being declared instead to have a different function such as that of a solubilizing excipient as in the case of cyclodextrins [38].

We report the list of carriers described in the IMPDs in Table 2.

Only 1 out of 433 CTs turned out to include in the CMC section of the IMPD a nano-related term, making explicit reference to the use of a nanotechnology. The only term identified was “nanobody”. Nanobodies, or variable domains of heavy chain-only antibodies (VHH), are single-domain antibody fragments with a molecular weight of about 14 kDa [39]. The origin of the name derives directly from the reduced size of the molecule. Nevertheless, we were not able to retrieve information on its characterization confirming dimensions on the nanometric scale.

No IMP was explicitly coded as a nanomedicine in the IMPD, or in the CTA; however, according to their characterization, for some of them, selected analytical methods were utilized such as dynamic light scattering or negative-stain transmission electron microscopy, and results confirm that one or more external dimensions are in the size range of 1–100 nm. However, as with the term “nanomedicines”, a wide variety of nanostructures or nanomaterials could be identified so far, each one with specific and peculiar attributes, and a case-by-case approach is acknowledged and indeed currently adopted by sponsors to analyze and characterize them, denoting a definitely not standardized approach. On the other hand, it is not enough to have an analytical method confirming a nanometer scale dimension to classify a nanostructure as a nanomedicine; what instead actually circumscribes a nanomedicine seems to be, rather, the structure and/or function. We therefore list in Table A1, among the CTs authorized in 2018, those IMPs with a nanoscale dimension confirmed and a relatively high number of IMPs that were identified, according to their description, as potential typical nanostructures or nanomaterials or with characteristics attributable to a nanomedicine, even if in the absence of a dedicated nano-related term or statement in the IMPD confirming the use of a nanotechnology, or a nanoscale dimension confirmed during the characterization.

**Figure 3:**
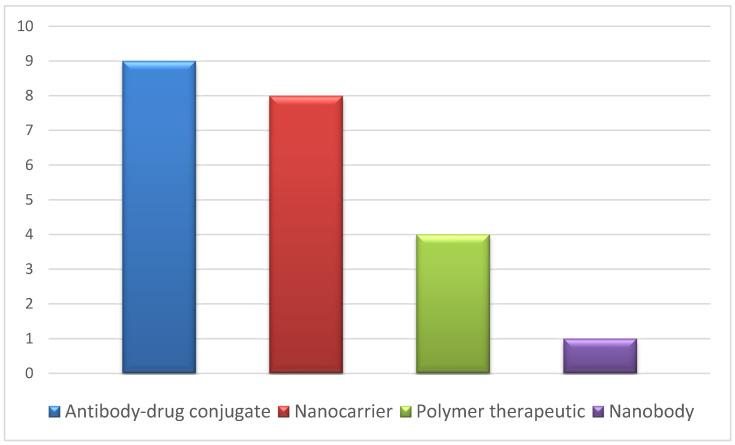
Categories of nanomedicines identified in the IMPDs and number of instances.

These IMPs were not explicitly coded as nanomedicines in the IMPD, or in the CTA; however, they may be classified into categories attributed to nanomedicines, such as nanocarrier, antibody–drug conjugate or polymer therapeutic, according to the JRC Technical Report [11]. We report in Figure 3 the different categories identified and the number of instances.

Depending on their characterization or control strategy, analytical methods such as electrospray ionization mass spectrometry, matrix-assisted laser desorption ionization time-of-flight, size exclusion high-performance liquid chromatography and sodium dodecyl sulfate-polyacrylamide gel electrophoresis were utilized. Specific parameters were tested, mainly the molecular weight, providing quantitative information about the molecular size with results that are potentially compatible with the nanometer scale, even though confirmation is actually not provided in the CMC quality documentation.

Monoclonal antibodies or other recombinant products such as fusion proteins were excluded from the scope, considering that, despite their sometimes high structural complexity, they are nevertheless single biological molecules. Chimeric antigen receptor T cells were also excluded due to their size, which exceeds the nanometric range. One group of products that may be, in a broad sense, counted in the field of nanomedicines are viral vectors. Viruses can indeed be seen as nanocarriers that can release their genetic cargo in the cell. The exterior of the virus can be conveniently functionalized so as to target specific cells [40]. Viral vectors in Table A1 are adenoviruses or adeno-associated viruses, whose dimensions were experimentally confirmed only in a few cases. Nevertheless, the size of this type of virus is in the nanometric scale. There is also a vaccinia virus vector, which is bigger than the others, exceeding the value of 100 nm, but the mode of action is essentially the same. Another group is represented by chemically modified proteins [41]. Antibody–drug conjugates can be considered nanomedicines in which the antibody is a carrier of nanometric dimensions and can target its payload directly into the cell after internalization. In our list, they are mostly constituted of a humanized monoclonal antibody of the IgG1 subclass, while the chemical counterpart is a cytotoxic compound. Instead, PEGylation can be seen as a chemical modification that masks the protein and alters its pharmacokinetics. Further, a PEGylated oligonucleotide is in our list and, in this case, PEG constitutes more than three quarters of the mass of the conjugate.

The IMPs listed in Table A1 are relatively simple compared to potentially more complex nanostructures, once again highlighting the gap between scientific research and clinical application. In general, no specific correlation or dedicated characterization was provided in terms of potential impact as a nanomedicine or nanocarrier on the IMP safety profile, and this highlights and poses additional reflections not only on the need to develop a specific guidance on nanomedicines in CTs but also to extend the concept to any nanostructure or nanomaterial associated with an IMP, including potential promising nanocarriers, whose molecular structure form clearly affects the size, stability, entrapment efficiency, pharmacokinetics, pharmacodynamics and targeting properties [42] and could imply additional or unknown safety risks.

If we rely on the information provided as structured data in the CTA, or even on performing a text search browsing quality documentation by keyword, it is not possible to discern if the IMPs qualify as a nanomedicine or not. If we go through the quality-related information on the IMPs in the CMC section of the IMPDs, we find limited to no information explicitly provided by the sponsors useful to the assessor at the CA in order to identify clearly and at a first glance a nanomedicine or an IMP-related nanostructure or nanomaterial. In addition, there is not a standardized approach to the characterization of a nanomedicine; in fact, analytical methods and parameters tested may be different for the same category of IMPs, and critical quality attributes (CQAs), as a consequence, may not be always fully addressed. A clear and unambiguous definition of nanomedicine in a CT’s regulatory framework is actually missing, and indeed the current applicable guidelines on the requirements for the chemical and pharmaceutical or biological quality documentation concerning IMPs in CTs [43,44] do not even mention or provide guidance on the assessment of quality requirements of nanotechnology-based IMPs. Sponsors should instead be able to report as structured data in section “D Information on each IMP” of the CTA if the IMP classifies as a nanomedicine (or nanodevice), and a discussion on proper characterization and quality attributes, including safety assessment of the IMP, should also be reported in the IMPD. It is also important to emphasize how not only the IMP’s nano-characteristics should be highlighted but also how the impurity profile could impact the safety, where, as an example, high-molecular weight dimers and aggregates or even lower-molecular weight degradants could be identified as a nano-related product’s impurities.

## 4. Regulatory Reflections

Databases and online CT registries do not allow retrieving accurate information on the actual use of surfactants, nanomedicines and nanocarriers in CTs authorized in the EU. An emerging issue identified is the missing structured information in the CTA form regarding nanomedicines (and nanodevices), or nanotechnology-based IMPs. The ability to search for a specific term in free text fields in an official CT register to have an estimation of the actual number of surfactants, nanomedicines and nanocarriers used in CTs is not sufficient. The only accurate process that could be applied today is to go through the quality documentation of each and every CT assessed and authorized by CAs, and to search for the keywords within the documentation, but this is a time-consuming and resource-expensive process, and, in particular, if we wanted to obtain the information for all the EEA MS, this would demand a huge effort and results would not be provided immediately. However, even when such a manual process is put in place, it is not always straightforward to retrieve consistent information from the CMC documentation, as surfactants, nanomedicines and nanocarriers could not be coded as such in the presented application dossier and quality documentation.

It is a challenging activity for the assessors at the NCAs to keep up to date with the latest scientific progresses and to constantly ensure the highest standards of professional training. Awareness sessions are organized by the EMA’s Innovation Task Force, but the field is evolving quickly, and additional efforts are needed to continue to support assessors in ensuring the safety of subjects and in enabling them to provide, in parallel, feedback and input to regulatory policy-makers in order to contribute to an always more open, transparent and evidence-based policy making, allowing driving regulatory progress towards a better regulation [45] process. It is, in addition, due to the high-speed innovation evolution that there is always the more frequent need to interact with other regulatory sectors traditionally not directly involved in drug development. ML, a fast-growing technology, and an application of artificial intelligence (AI), is as an example identified as one of the relatively recent and near-future most important drivers of advances [46] in target identification of drug discovery and development [47]. The need for a structured approach to a constant scientific update and interdisciplinary collaboration among regulators and with the scientific community is therefore a growing need acknowledged by regulators. Nanoscience and nanotechnology are among the most advanced frontiers of innovation along with AI and ML applied technologies that are already and will for sure in the near-future continue to impact IMPs’ and CTs’ evolution. Should any urgent safety issue arise, potentially linking to surfactants, nanomedicines (or nanodevices) and nanocarriers and, in general, to nanotechnology-based IMPs tested in CTs, regulators would need to be able to immediately identify those potential risks and envisage any class enlarged effect to protect and safeguard public health. This is why dedicated structured data fields are required to be foreseen in the registers, in order to specify in the description of the IMP if a nanomedicine (or nanodevice) criterion applies, in addition to clarifying if there is any nanostructure or nanomaterial in the IMP tested in the CT by declining the corresponding category. Once the Regulation (EU) No 536/2014 becomes applicable, it would be a proactive approach and a great chance in the EU to support the implementation of the first CT register with a highly effective level of details in terms of description of the IMP, unique worldwide, that might acknowledge and keep up with the latest technologies and innovations with such a level of deep stratified information in order to support safety oversight. This would be possible and in line with the functional specifications for the EU portal and EU database to be audited [48], where it is clearly stated that the sponsor needs to be able to submit information related to a new IMP and/or a new substance through the EU portal.

In terms of transparency, there is an additional reflection to be made by the scientific community and by regulators: information on CTs and IMPs under test is not always fully accessible and, when accessible, its availability is not straightforward, particularly in the EEA. When this is available, in fact, there is no standard process in place to share information transparently with the scientific community, scientists or academia, and even when structured data are available in databases, there is often no functionality that helps in retrieving them. Even when data are available for the NCA, they are not easy to retrieve and analyze unless a manual process is put in place. An adequate level of scrutiny and transparency in CT-related information is crucial to ensure the protection of public health and to foster, at the same time, innovation in the medical research field with the purpose of supporting early access to the best treatment for patients, including personalized medicine. However, there is a lack of data access to some aspects of CTs, partially due to the intrinsic nature of the information in the CT application such as the protection of commercially confidential information. However, also in this case, a stratified and anonymized set of information could nonetheless be provided, enabling the possibility to also identify trends, but, of course, only as far as if data access were to be feasible and the competent authorities were to have resources and proper information technology tools to work with. As previously reported, the lack of transparency may also be due to the need to improve compliance as recently stressed in the Joint Letter by the European Commission, EMA and Heads of Medicines Agencies. Summaries of results of concluded CTs should be publicly available in the EU CTs database within one year (6 months for pediatric trials) after the end of a CT. Increased transparency in the area of CTs is one of the reasons for the proposal for a Regulation repealing Directive 2001/20/EC/. Further, there is an increasing number of areas of CTs that are becoming very important due to technology and science progress, such as nanotechnology and the use of AI that were not identified in the past as potentially related to CTs and therefore were not coded within an application, but that are already today and for sure in a more deep fashion in the near-future going to pervade and spread across all sectors of CTs, potentially changing or expanding regulatory boundaries.

However, how can regulators manage this challenge if those aspects and data are not properly coded in a CT application? Currently, there are no structured data where the sponsor actually declares that the IMP is a nanomedicine or a nanodevice or has a nanostructure or nanomaterial component, or that AI or ML technology is being used to support or manage the CT. This poses a potential risk when assessing parameters of the quality, efficacy and safety of a specific treatment including pharmacokinetics parameters (i.e., absorption, distribution, metabolism and excretion), which can change depending on the specific nanotechnology used or on the big data and ML technique used to estimate the risk of adverse reactions and to assess the safety and efficacy and benefit/risk ratio. An added value would definitely be the inclusion, in the EU portal and database, as foreseen in article 80 and article 81 of the Regulation (EU) No 536/2014, in the CT application and, in addition to those fields already currently available in section “D.3 Description of the IMP”, in the current CTA, according to the Annex 1 Clinical Trial Application Form of the Directive 2001/20/EC, of structured data fields for the collection of more detailed information on the IMP and, in particular, of specifying whether the IMP is a nanomedicine, if it includes a nanodevice or if there is any nanotechnology-based IMP.

The next step would be providing a standardized definition and coding within a regulatory framework of all possible nanomedicines, nanotechnology-based IMPs, nanostructures and nanomaterials and the elaboration of additional dedicated guidelines applicable to CTs in order to support the submission and assessment of CQAs. Additional guidelines from the EMA would be highly recommended as a support tool in order to ensure the appropriate citation and usage of nanotechnologies and surfactants. This reflects a still missing harmonization in the current European regulatory framework and in the supervision of nanomaterials in healthcare products [49]. Reflection papers are available such as those on intravenous medicinal products containing active substances solubilized in micellar systems (non-polymeric surfactants) and on development of block-copolymer micelle products [50,51]. Their validity is not questioned; however, it must be recognized that given the complexity of micelle systems, e.g., when delivery in addition to a solubilizing function is involved, special properties affecting kinetics and distribution in vivo may not be clearly reported in the IMPD, and therefore the elaboration of additional guidelines could definitely be of support. A strong pharmaceutical development is anyhow needed including development and validation of some specific tests with the purpose to understand what happens to the product after administration and to provide information on the system state in vivo, such as pharmacokinetic and pharmacodynamic properties that may impact on safety and efficacy.

## 5. Conclusions

With this research, we focused on a few terms, providing an objective analysis on the current actual use of surfactants, nanomedicines and nanocarriers in CTs with reference to those authorized in one regulatory authority in the EU, the Italian Medicines Agency in 2018, also providing an estimation of their use across the EEA, and contributing to transparency, thus enlarging the access to relevant data. Results highlight potential indications and inputs for further reflections on actions for regulators to take into considerations, in order to actively and safely drive nanotechnology innovation in CTs.

A universally accepted definition of nanomedicine is missing and there is limited availability of guidelines supporting its submission and assessment in the context of CT applications; moreover, no structured data are available in the CTA form to collect detailed information on nanotechnology-based IMPs. The regulatory framework leaves margins of freedom, and a lack of guidance is noted for the provision of quality information in the CMC section of the IMPDs for nanomedicines or nanotechnology-based IMPs, potentially not fully addressing all CQAs and issues on the safety profile. CQAs are indeed not clearly identified and defined for nanomedicines and nanotechnology-based IMPs in the CMC documentation submitted as part of an application for authorization of CTs, and analytical methods proposed by sponsors are not standardized also considering the variability of nanostructures or nanomaterials and their function in the IMP composition. A new impulse should be given to the optimization of dedicated guidelines on nanotechnology-based IMPs, and additional efforts should be made in providing clarifications regarding nanomedicine and related term definitions in a CT’s regulatory framework.

Standard surfactants are used in CT Phases I–III with no evidence of any dedicated use across the study phases or therapeutic areas or formulation of the IMP. No use of any novel surfactant excipient was identified, and polysorbates were the standard surfactants used, together accounting for more than 82% of the cases. This is understood as an approach to foster the CT approval process, proposing surfactants for whose pharmacopoeia standards are available. On the other hand, this limits the translation of innovative nanotechnology into a clinical path. Regulatory support for the use of innovative excipients should be envisaged and a constructive early-phase dialogue should be established capitalizing on available procedures such as scientific advice, and the support and guidance provided by the EU-Innovation Network (EU-IN). It is, however, noted that there is a missing direct link between scientific research, pharmaceutical development and regulatory environments, where additional collaboration would result in promotion of innovative solutions, allowing sponsors of CTs to test them in early development phases.

No evidence was retrieved about the use in CTs of structured delivery systems by chemical entities such as non-ionic surfactant-based nanocarriers (niosomes, nanoemulsions, micelles, etc.). The use of nanocarriers in CTs is limited and mainly only related to active substances of biological or biotechnological origin. Information on nanocarriers is not available in the CTA form and in the related quality information in the CMC section of the IMPDs; however, when nanocarriers are identified, they are not explicitly coded as such and their characterization is not standardized.

## Figures and Tables

**Table 1:** Surfactants described in the Investigational Medicinal Product Dossiers (IMPDs).

Surfactant	Function	Study Phases	Therapeutic Areas (Number of Instances)	% of Use
Lauroyl polyoxyl-32 glycerides (Gelucire 44/14)	Surfactant	II	Physiological processes (1)	0.49
Mannide monooleate	Emulsifier	I	Cancer (1)	0.49
Sodium lauryl sulfate	Surfactant	III	Blood and lymphatic diseases (1) Cancer (9) Immune system diseases (1) Musculoskeletal diseases (2) Nervous system diseases (1)	6.86
Polyoxyl 40 hydrogenated castor oil	Surfactant	III	Blood and lymphatic diseases (2) Cancer (1) Immune system diseases (1)	1.96
Poloxamer 188 (Pluronic F-68)	Surfactant	I/II/III	Blood and lymphatic diseases (2) Cancer (1) Eye diseases (2) Immune system diseases (3) Nervous system diseases (2) Nutritional and metabolic diseases (1) Respiratory tract diseases (1)	5.88
Poloxamer 407	Surfactant Plasticizer	I/II	Cancer (1) Nervous system diseases (1)	0.98
Polysorbate 20 (Tween 20)	Surfactant Stabilizing agent	I/II/III	Bacterial infections and mycoses (1) Blood and lymphatic diseases (1) Cancer (45) Cardiovascular diseases (3) Digestive system diseases (4) Eye diseases (6) Immune system diseases (5) Nervous system diseases (5) Nutritional and metabolic diseases (1) Virus diseases (4)	36.76
Polysorbate 80 (Tween 80)	Surfactant Stabilizing agent	I/II/III	Digestive system and oral physiological phenomena (2) Metabolic phenomena (1) Blood and lymphatic diseases (5) Cancer (57) Cardiovascular diseases (2) Congenital, hereditary and neonatal diseases and abnormalities (1) Digestive system diseases (5) Eye diseases (1) Immune system diseases (7) Nervous system diseases (8) Nutritional and metabolic diseases (1) Respiratory tract diseases (1) Skin and connective tissue diseases (3)	46.08
Propylene glycol monocaprylate, type I (Capryol PGMC)	Surfactant	II	Physiological processes (1)	0.49

**Table 2:** Carriers described in the IMPDs.

Code	Description	Term in IMPD	Pharmaceutical Form	Study Phase	Therapeutic Area	Active Substance of Chemical Origin?	Active Substance of Biological/Biotechnological Origin?	Gene Therapy Medicinal Product?
C1	Human serum albumin (HSA)	Carrier protein	Solution for infusion	II	Eye diseases	No	Yes	No
C2	Keyhole limpet hemocyanin (KLH) subunits	Carrier protein	Suspension for injection	II	Nervous system diseases	No	Yes	No
C3	Red blood cells (RBC)	Carrier	Solution for infusion	III	Cancer	No	Yes	No
C4	Non-toxic mutant of diphtheria toxin (CRM197)	Carrier	Suspension for injection	III	Bacterial infections and mycoses	No	Yes	No
C5	mPEG succinimide	Carrier	Powder and solvent for solution for injection	II	Hormonal diseases	No	Yes	No
C6	Tetanus toxoid protein	Carrier protein	Solution for injection	III	Bacterial infections and mycoses	No	Yes	No
C7	Lactose α-monohydrate	Carrier	Inhalation powder	III	Respiratory tract diseases	Yes	No	No
C8	Copovidone	Carrier polymer	Coated tablet	III	Cancer	Yes	No	No
C9	Sugar spheres	Carrier	Capsules	III	Immune system diseases	Yes	No	No
C10	Vitamin E polyethylene glycol succinate	Carrier	Capsules	II	Cancer	Yes	No	No

## Data Availability

Additional information on the data presented in this study are available on request from the corresponding author. The data are not publicly available due to protection of commercially confidential information.

## Supplementary Materials

The following are available online at https://www.mdpi.com/1999-4923/13/3/381/s1, Table S1: Chemical structures of surfactants reported in Table 1.

## Appendix A

**Table A1:** Nanomedicines in the IMPDs.

Code	Description	Nanomedicine-Related Term	Analytical Method Confirming Nanoscale Dimension	Pharmaceutical Form	Study Phase	Therapeutic Area	Active Substance of Chemical Origin?	Active Substance of Biological/Biotechnological Origin?	Gene Therapy Medicinal Product?
N1	Serotype 5 adenovirus vector	Nanocarrier	Yes	Concentrate for solution for injection	I/II	Cancer	No	No	Yes
N2	Adeno-associated virus serotype 8 gene therapy vector	Nanocarrier	Yes	Solution for injection	I/II	Blood and lymphatic diseases	No	No	Yes
N3	Monoclonal antibody conjugated to a DNA alkylating agent through a linker	Antibody–drug conjugate (ADC)	No	Powder for solution for infusion	I	Cancer	No	Yes	No
N4	Adeno-associated virus serotype 9 vector	Nanocarrier	No	Solution for infusion	III	Nervous system diseases	No	Yes	Yes
N5	Adeno-associated virus serotype 8 gene therapy vector	Nanocarrier	No	Concentrate for solution for infusion	I/II	Nutritional and metabolic diseases	No	No	Yes
N6	Recombinant adeno-associated viral vector	Nanocarrier	Yes	Concentrate for solution for infusion	II	Congenital, hereditary and neonatal diseases and abnormalities	No	No	Yes
N7	PEGylated enzyme	Polymer therapeutic	No	Solution for injection	III	Genetic phenomena	No	Yes	No
N8	Monoclonal antibody conjugated to a prodrug through a linker	Antibody–drug conjugate (ADC)	Yes	Powder for solution for infusion	III	Cancer	Yes	Yes	No
N9	Monoclonal antibody conjugated to an antimitotic agent through a linker	Antibody–drug conjugate (ADC)	No	Powder for solution for infusion	III	Cancer	No	Yes	Yes
N10	Recombinant adeno-associated virus vector serotype 2	Nanocarrier	No	Suspension for injection	III	Eye diseases	No	No	Yes
N11	Monoclonal antibody conjugated to an antimitotic agent through a linker	Antibody–drug conjugate (ADC)	No	Powder for solution for injection	I	Cancer	No	Yes	No
N12	Monoclonal antibody conjugated to a cytotoxic agent through a linker	Antibody–drug conjugate (ADC)	No	Powder for solution for infusion	I	Cancer	No	Yes	No
N13	Vaccinia virus vector	Nanocarrier	No	Solution for injection	I/II	Virus diseases	No	Yes	Yes
N14	Adeno-associated virus serotype 5 vector	Nanocarrier	Yes	Solution for infusion	III	Blood and lymphatic diseases	No	No	Yes
N15	Monoclonal antibody conjugated to a cytotoxic agent through a linker	Antibody–drug conjugate (ADC)	No	Powder for solution for infusion	III	Cancer	Yes	Yes	No
N16	Trivalent Nanobody	Nanobody	No	Nebulizer solution	II	Virus diseases	No	Yes	No
N17	Pegylated peptide	Polymer therapeutic	No	Solution for injection	II	Blood and lymphatic diseases	Yes	No	No
N18	Glycopegylated recombinant protein	Polymer therapeutic	No	Powder and solvent for solution for injection	III	Congenital, hereditary and neonatal diseases and abnormalities	No	Yes	No
N19	Monoclonal antibody conjugated to a cytotoxic agent through a linker	Antibody–drug conjugate (ADC)	No	Solution for infusion	II	Cancer	No	Yes	No
N20	Pegylated oligonucleotide	Polymer therapeutic	No	Solution for injection	II	Eye diseases	Yes	No	No
N21	Monoclonal antibody conjugated to an antimitotic agent through a linker	Antibody–drug conjugate (ADC)	No	Solution for infusion	II	Cancer	Yes	Yes	No
N22	Monoclonal antibody conjugated to an antimitotic agent through a linker	Antibody–drug conjugate (ADC)	No	Powder for solution for infusion	II	Cancer	No	Yes	No
