# Supplementary material for: Surfactants, Nanomedicines and Nanocarriers: A Critical Evaluation on Clinical Trials
Source: Pharmaceutics. 2021 Mar 13;13(3):381. doi: 10.3390/pharmaceutics13030381 (PMC7999832; doi:10.3390/pharmaceutics13030381)
Supplement: Supplementary file 1 [file pharmaceutics-13-00381-s001.pdf]

pharmaceutics

MDPI

Supplementary Materials: Surfactants, Nanomedicines and Nanocarriers: A Critical Evaluation on Clinical Trials

Diego Alejandro Dri, Carlotta Marianecci, Maria Carafa, Elisa Gaucci and Donatella Gramaglia

Table S1. Chemical structures of surfactants reported in Table 1.

<table>
<tr><th>Surfactant</th><th colspan="3">Structure</th></tr>
<tr><td>Lauroyl polyoxyl-32 glycerides
(Gelucire 44/14)</td><td colspan="3">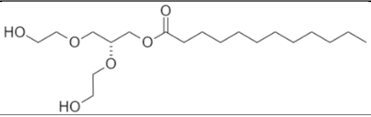</td></tr>
<tr><td>Mannide monooleate</td><td colspan="3">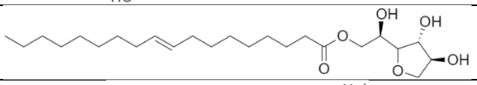</td></tr>
<tr><td>Sodium lauryl sulfate</td><td colspan="3">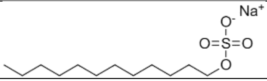</td></tr>
<tr><td>Polyoxyl 40 hydrogenated castor oil</td><td colspan="3">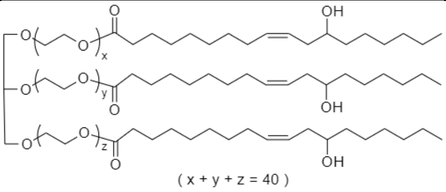</td></tr>
<tr><td rowspan="5">Poloxamer 188
(Pluronic F-68)

Poloxamer 407</td><td colspan="3">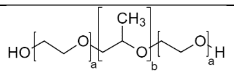</td></tr>
<tr><td colspan="3">The label states the type of poloxamer</td></tr>
<tr><td>Poloxamer Type</td><td>Ethylene Oxide Units a</td><td>Propylene Oxide Units b</td></tr>
<tr><td>188</td><td>75–85</td><td>25–30</td></tr>
<tr><td>407</td><td>95–105</td><td>54–60</td></tr>
<tr><td>Polysorbate 20
(Tween 20)</td><td colspan="3">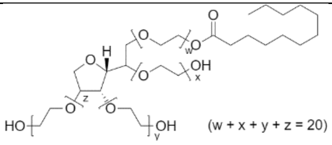</td></tr>
<tr><td>Polysorbate 80
(Tween 80)</td><td colspan="3">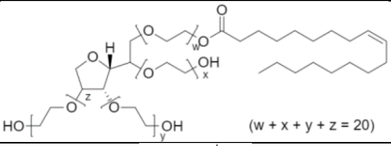</td></tr>
<tr><td>Propylene glycol monocaprylate, type I
(Capryol PGMC)</td><td colspan="3">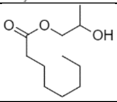</td></tr>
</table>
